# ILC3 function as a double-edged sword in inflammatory bowel diseases

**DOI:** 10.1038/s41419-019-1540-2

**Published:** 2019-04-08

**Authors:** Boning Zeng, Shengnan Shi, Gareth Ashworth, Changjiang Dong, Jing Liu, Feiyue Xing

**Affiliations:** 1 Institute of Tissue Transplantation and Immunology, Department of Immunobiology, Jinan University, Guangzhou, China; 2 Key Laboratory of Functional Protein Research of Guangdong, Higher Education Institutes, Jinan University, Guangzhou, China; 3 School of Stomatology, Jinan University, Guangzhou, China; 4 BioMedical Research Centre, University of East Anglia, NR4 7TJ Norwich, UK

## Abstract

Inflammatory bowel diseases (IBD), composed mainly of Crohn’s disease (CD) and ulcerative colitis (UC), are strongly implicated in the development of intestinal inflammation lesions. Its exact etiology and pathogenesis are still undetermined. Recently accumulating evidence supports that group 3 innate lymphoid cells (ILC3) are responsible for gastrointestinal mucosal homeostasis through moderate generation of IL-22, IL-17, and GM-CSF in the physiological state. ILC3 contribute to the progression and aggravation of IBD while both IL-22 and IL-17, along with IFN-γ, are overexpressed by the dysregulation of NCR^−^ ILC3 or NCR^+^ ILC3 function and the bias of NCR^+^ ILC3 towards ILC1 as well as regulatory ILC dysfunction in the pathological state. Herein, we feature the group 3 innate lymphoid cells’ development, biological function, maintenance of gut homeostasis, mediation of IBD occurrence, and potential application to IBD therapy.

## Facts

- Group 3 innate lymphoid cells (ILC3) are responsible for gastrointestinal mucosal homeostasis through moderate generation of IL-22, IL-17 and GM-CSF in the physiological state.
- ILC3 contribute to the progression and aggravation of inflammatory bowel diseases by the dysregulation of NCR^−^ ILC3 or NCR^+^ ILC3 function and the bias of NCR^+^ ILC3 towards ILC1 under the stimulation of IL-12 generated by CD14^+^ dendritic cells as well as regulatory ILC dysfunction in the pathological state.
- The dysregulation of ILC3 results in overexpressions of inflammatory cytokines IL-22, IL-17 and IFN-γ, in which IL-17 can recruit neutrophil cells to disrupt E-cadherin and junctional adhesion molecule-like molecule (JAML), leading to the enhancement of epithelial permeability.
- The ILC3 to ILC1 plasticity is reversible in the presence of IL-23, IL-1β and retinoic acid produced by CD14^−^ dendritic cells.

## Open questions

- What is an exact mechanistic process of the ILC3 dysregulation?
- Whether does the ILC3 dysregulation directly impact ILCreg?
- How to reverse the ILCreg dysfunction and control the level of CD14 DC?
- Why do the antibodies against human IL-17A or IFN-γ not show significant efficacy in the treatment of patients with Crohn’s disease?

## Introduction

Inflammatory bowel diseases (IBD) display non-specific and chronic inflammatory lesions occurring in the intestinal mucosa and submucosa, represented by ulcerative colitis (UC) and Crohn’s disease (CD) that are considered distinct entities^1^. Although inherited tendency, environmental factors, microbial infection and inappropriate immune responses are, up to now, considered to be relevant, detailed etiology and pathology of the diseases are still obscure, and the existing remedies are not satisfactory. Moreover, the risk of other chronic diseases or even colorectal cancer (CRC) is dramatically increased in patients suffering from IBD. Studies indicate that chronic inflammation is a leading factor that converts low and high-grade dysplasia into CRC, and about 10–15% of the CRC patients die from IBD^2^.

Although patients with CD and UC have some similar pathological alteration, clinical symptoms and signs, there are still some differences between them. CD can intrude upon one or more regions of the intestinal mucosa and submucosa, but the terminal ileum and colon are mainly implicated. In contrast, UC has a significant impact on the mucosal layer of the colon or rectum with enduring inflammation and ulcers^3^. Studies have shown that CD is mediated by a Th1 response. The highly expressed-tumor necrosis factor (TNF), interferon-γ (IFN-γ) and interleukin-17A (IL-17A) were found in CD4^+^ T cells separated from inflammatory mucosal areas. Conversely, UC is mediated by a Th2 immune response as IL-4, IL-5, and IL-13 are highly secreted in inflamed tissue^4^. Genome-wide associated studies and meta-analyses have identified 200 loci related to both CD and UC^5,6^, showing that most of the identified genes are associated with other autoimmune diseases, such as psoriasis^7^. Interestingly, approximately 70% of IBD-related genes are identical between CD and UC, while only 23 and 30 loci are specifically related to the sensitivity to UC and CD, respectively^5,8^. These susceptible genes are mainly linked to the host immune system, including adaptive and innate immune responses to protect epithelial tissue from mycobacteria and autophagy^9^. In recent years, innate immune cells (ILC) and their interaction with inflammatory bowel diseases have attracted broad attention. Here, we focus on mechanistic processes of ILC3 action in development of IBD.

## Innate lymphoid cells

**Fig. 1:**
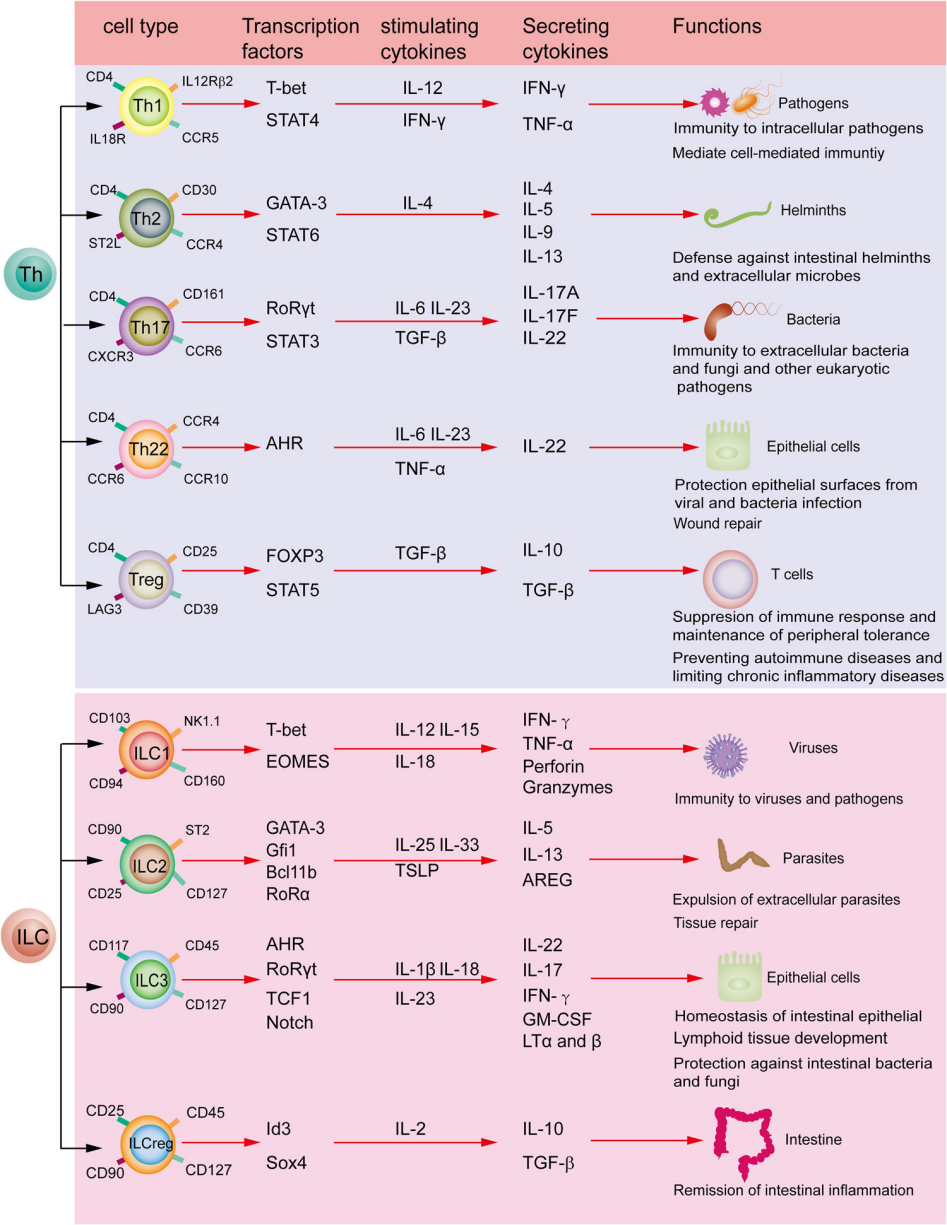
Characteristic comparison of ILCs with Th cells. According to different cellular phenotypes, transcriptional factors and functional factors, T helper (Th) cells can be classified into Th1, Th2, Th17, Th22, Treg and so on. By contrast, ILCs can be divided into three groups ILC1, ILC2, ILC3 and ILCreg

ILC develop from common lymphoid progenitor cells (CLP) and show resemblance to adaptive lymphocytes in morphology. Dissimilarly, ILC have no rearranged antigen-specific receptors but CD127 (IL-7Rα) is enriched on their surface^10^. They can be divided into three groups on the basis of expression of transcriptional factors and cytokines^11^. Group 1 ILC (ILC1) can secrete the Th1-like cytokine interferon-γ (IFN-γ) under the stimulation of IL-12, IL-15, and IL-18 in the presence of transcriptional factor T-bet^12,13^. Group 2 ILC (ILC2) can generate Th2-like cytokines IL-5 and IL-13 under the stimulation of IL-25 and thymic stromal lymphopoietin (TSLP) in the presence of transcriptional factors RoRα and GATA3^14^. Group 3 ILC (ILC3) produce Th17- and Th22-like cytokines IL-22, IL-17, and a granulocyte macrophage-colony stimulating factor (GM-CSF) under the stimulation of IL-23 and/or IL-1β in the presence of the RORγt and aryl hydrocarbon receptor (AHR)^15^. A novel recognized subset is regulatory ILC that secrete IL10 and TGF-β under the stimulation of IL-2 in the presence of transcriptional factors Id3 and sox4^16^. These classification features look similar to those of CD4^+^ T helper cell subsets (Fig. 1). Recently, ILC have increasingly been considered as a key moderator of tissue homeostasis and inflammation *via* releasing cytokines. They have been known to exhibit protective responses against microorganisms in lymphoid tissue formation and in tissue remodeling after damage^17^.

Analogous to T and B cells, ILC are developed from CLP. In the presence of nuclear factor interleukin-3 and inhibitor of DNA-binding 2 (Id2), CLP deviate into restricted common helper-like innate lymphoid progenitor cells (CHILP). Afterwards, downstream precursor cells (ILCP) of ILC express transcription factor PLZF and can give rise to the ILC1, ILC2 and ILC3 subsets^18,19^. Accumulating evidence shows that RORγt (encoded by Rorc) drives differentiation of ILC3 from their precursor ILCP^20^. The common cytokine receptor γ-chain (γc) is essential for the maturation of ILC in mice, constituting the components of IL-2, IL-4, IL-7, IL-9, IL-15, and IL-21^21^. IL-15 is an indispensable regulator for the development and differentiation of ILC1 and natural killer (NK) cells. In contrast, ILC2 and ILC3 are dependent on IL-7 for development. Knocking out IL-7 or IL-7Rα will result in a greatly reduced number of ILC2 and ILC3^22^. However, the precise differentiation and regulatory mechanisms of ILC in humans and mice remain elusive.

Group 1 ILC comprise of NK cells and ILC1, they are primarily involved in eliminating viruses and intracellular pathogens. Both NK cells and ILC1 secrete IFN-γ and depend on the transcriptional factor T-bet. NK cells express transcriptional factor Eomesodermin (Eomes), which is a key factor discriminating them from ILC1^23^. They express Nkp46, NK1.1 and CD90 in mice, but CD56, CD16 and CD94 in humans^24^. Apart from NK cells, ILC1 may be further categorized to two subsets of Group 1 ILC, lamina propria ILC1 and intraepithelial ILC1 (iILC1) (Table 1). In mice, lamina propria ILC1 produce CD127, Nkp46, T-bet and CD161, and iILC1 yield CD103, CD160, and NK1.1^25^. In humans, lamina propria ILC1 generate CD127 and CD161, but not NKp44, CD56 and c-kit. In contrast, iILC1 express CD56, CD103, CD94, CD160 and Nkp44, but lack CD127^25,26^. ILC1 are mainly distributed within the intestinal lamina propria in both mice and humans, and the proportion of ILC1 is notably increased in IBD patients^27^.

**Table 1:** Characteristics of innate lymphoid cells in mice and human

ILC Group	Phenotype		Localization	Disease	Reference
	mouse	Human			
Group1 ILC					
NK Cell	Lin^−^, Eomes^+^, NK1.1^+^,NKP46^+^, T-bet^+^, CD90^+^, CD127^−^	Lin^−^, CD127^−^, CD117^−^, CD25^+^,CD16^+^, CD56^+^, CD94^+^, CD161^+^	Spleen Lymph node	Crohn’s disease	^23, 24, 95^
Intraepithelial ILC1	Lin-, NK1.1^+^, CD103+,CD160^+^, CD127^−^	Lin^−^, Nkp44^+^, CD103^+^,CD127^−^, CD160^+^, CD94^+^	Intestine lamina propria, Intestine epithelial layer	Crohn’s disease Colitis	^25, 26, 28^
Lamina propria ILC1	Lin^−^, Nkp46^+^, Tbet^+^, CD127^+^,CD161^+^	Lin^−^, CD56^−^, C-kit^−^, Nkp44^−^, CD127^+^, CD161^+^	Intestine, lamina propria	Crohn’s disease Colitis	^12, 25– 28^
Group2 ILC					
ILC2	Lin^−^, CD25^+^, SCA1^+^, ICOS^+^, ST2^+^, Thy1^+^, IL7Rα^+^, CD117^+^	Lin^−^, CD161^+^, ST2^+^, CRTH2^+^,CD127^+^, NK1.1^+^,Nkp44^−^, CD25^+^,CD117^+^, CD90^+^	Skin, lung, adipose tissue, spleen, MLN	Asthma Allergy Colitis	^14, 28, 29, 96^
Group3 ILC					
LTi	Lin^−^, CCR6^+^, CD4^+^, CD3^−^, CD45^+^,CD90^+^, CD117^+^, CD127^+^, IL-23R^+^	Lin^−^, CCR6^+^, CD45^+^, CD90^+^,CD117^+^, CD127^+^, CD4^−^, CD56^−^	Lymph node, Peyer’s patches	Autoimmune disease	^28, 32, 33^
NCR^−^ ILC3	Lin^−^, Nkp46^−^, CD25^+^, D90^+^,CD127^+^, CD117^−^	Lin^−^, CCR6^+^, Nkp44^−^, CD25^+^, CD117^+^, CD127^+^, CD161^+^	Epithelial tissues, Intestine, Skin	Crohn’s disease colitis	^28, 38, 41, 97^
NCR^+^ ILC3	Lin^−^, Nkp46^+^, CD117^+^, CD127^+^,CD90^+^	Lin^−^, CCR6^+^, Nkp44^+^, KP30^+^, CD117^+^, CD127^+^, CD161^+^	Epithelial tissues, Intestine Skin	Colitis	^24, 28, 39– 41^
Group4 ILC					
ILCregs	Lin^−^, CD45^+^, CD25^+^, D90^+^, CD127^+^, IL10^+^, CD4^−^, Foxp3^−^	Lin^−^, CD45^+^, CD25^+^, CD90^+^, CD127^+^, IL10^+^,CD4^−^, Foxp3^−^	Intestine	Colitis	^16^

*Lin*^*−*^ lineage marker-negative, *CRTH2* chemoattractant receptor-homologous molecule expressed on TH2 cells, *ICOS* inducible T cell co-stimulator, *CCR* CC-chemokine receptor, *SCA-1* stem cells antigen-1, *NCR* natural cytotoxicity receptor, *IL7Rα* CD127, *MLN* mesenteric lymph nodes

Group 2 ILC were first found in fetal gut tissue and later in skin, adipose tissue, lung, intestinal submucosa and lymphoid organs. ILC2 enable tissue to repair and defend against helminth infection^28^. They express IL7Rα, CD90.2 (Thy1), CD25, CD117, Sca1, KLRG1, ICOS and ST2 in mice, but CD25, CD90, CD127, CD161, ST2 and CRTH2 in humans^29^.

Group 3 ILC are a heterogeneous group, which can be divided into three subpopulations on the basis of their function during embryogenesis and their cell-surface expression of the natural cytotoxicity receptor NKp46^30^. Group 3 ILC are characterized by expressing transcription factors T-bet and RORγt and surface marker CD117 (c-kit)^31^. Lymphoid tissue inducer (LTi) cells were the earliest discovered ILC3, which facilitate the formation of lymph nodes and Peyer’s patches^32^. In mice, LTi cells express CD117, CD45, CCR6, CD4 and CD127, whereas human LTi cells show resemblance to mouse LTi cells, but do not produce CD4^33^. LTi cells assist the formation of lymphoid organs in response to TNF-α and lymphotoxin-β stimulation during embryogenesis. Postnatally, they can also produce IL-17A and IL-22 to defend the gastrointestinal tract against pathogens^34^. ILC3 exist mainly in the intestinal mucosal tissue, playing an important role in mucosal homeostasis and inflammatory responses. In addition to LTi cells, human ILC3 can also be subdivided into NCR^+^ ILC3 and NCR^−^ ILC3 in light of their expressing the natural cytotoxicity receptors NKp46, NKp44 and NKp30^35^. NCR^+^ ILC3 account for about 70% of the entire intestinal tract’s ILC. By contrast, NCR^−^ ILC3 are only around 15%^12,36^. In line with expressing chemokine CCR6, ILC3 can fall into CCR6^+^ LTi and CCR6^−^ ILC3 lineage^30,37^. NKp44 expresses on CCR6^−^ ILC3 in humans, but instead NKp46 in mice, which causes NCR^−^ ILC3 to eventually develop into NCR^+^ ILC3 in the presence of IL-1β plus IL-23 in vitro^26,38^. In mice, NCR^+^ ILC3 generate CD117, CD127 and Nkp46, but human NCR^+^ ILC3 instead highly express Nkp44 alongside a low level of Nkp46. Mouse NCR^−^ ILC3 are identical to human NCR^−^ ILC3 except that CD117 appears on human NCR^−^ ILC3 and not on mouse NCR^−^ ILC3^39^. NCR^+^ ILC3 primarily express IL22, but less IL-17. In contrast, NCR^−^ ILC3 predominantly produce IL-17, but a lesser amount of IL22^40^. In vitro, NCR^−^ ILC3 can switch to NCR^+^ ILC3 in the presence of IL-1β and IL-23^41^. RORγt and AHR are necessary for the development of CCR6^+^ and CCR6^–^ ILC3. A knockout of RORγt completely halts ILC3 formation, rather than that of ILC1 or ILC2. In addition, AHR highly expresses in ILC3 and is essential for maintaining ILC3^42^. However, the underlying mechanisms of ILC3 development and maintenance are not still elucidated.

Recently, Wang et al. identified a novel subset of ILC, named regulatory ILC (ILCreg). They found an IL-10-producing subset of innate cells that mainly presents in the intestinal tract and expresses various phenotypic ILC markers, such as CD25, IL-2R, Sca-1 and CD90. Furthermore, these cells express neither CD4 nor FoxP3, signature markers of CD4^+^ regulatory T cells (Treg), which makes ILCreg distinct from Treg. ILCreg are derived from CHILP instead of ILCP and highly express the transcriptional factors Id3 and Sox4, lacking other transcriptional factors that are essential for the development of ILC, such as Nfil3, Ror, Gata3 and AHR. ILCreg play an important role in innate immune responses, relieving intestine inflammation through generating IL-10 and TGF-β^16,43^. Interestingly, they resemble lymphoid cells morphologically with a high nuclear to cytoplasmic ratio. In addition, another regulatory subset, CD56^+^CD3^−^ ILC were recently recognized to display regulatory roles in human and mice. These cells express NK cell- and ILC-associated molecules, such as CD56, CD94, NCR3 (NKP30) and NCR1 (NKP46). Like ILCreg, the CD56^+^CD3^−^ ILC do not express Foxp3, but highly yield EOMES, TBX21, GATA3, RORA, and AhR. Dissimilarly, the CD56^+^CD3^−^ ILC may originate from NK cells, generate IL-22 but not IL10, and inhibit tumor-infiltrating lymphocytes^43,44^.

## ILC3 in the maintenance of gut homeostasis

ILC3 are implicated in gastrointestinal immune responses. They protect the intestinal mucosa from infections of various pathogens to maintain intestinal homeostasis in the steady state. This protective effect is mainly realized through the secretion of IL22, IL-17 and GM-CSF, triggering epithelial cells to produce antimicrobial peptides (AMPs), such as RegIIIβ and RegIIIγ that kill pathogens^45^, regulating T cell responses to commensal bacteria through the expression of a MHC-II molecule^46,47^, supporting the tolerance function of intestinal dendritic cells (DC) *via* GM-CSF secretion, and adjusting epithelial glycosylation^48^.

**Fig. 2:**
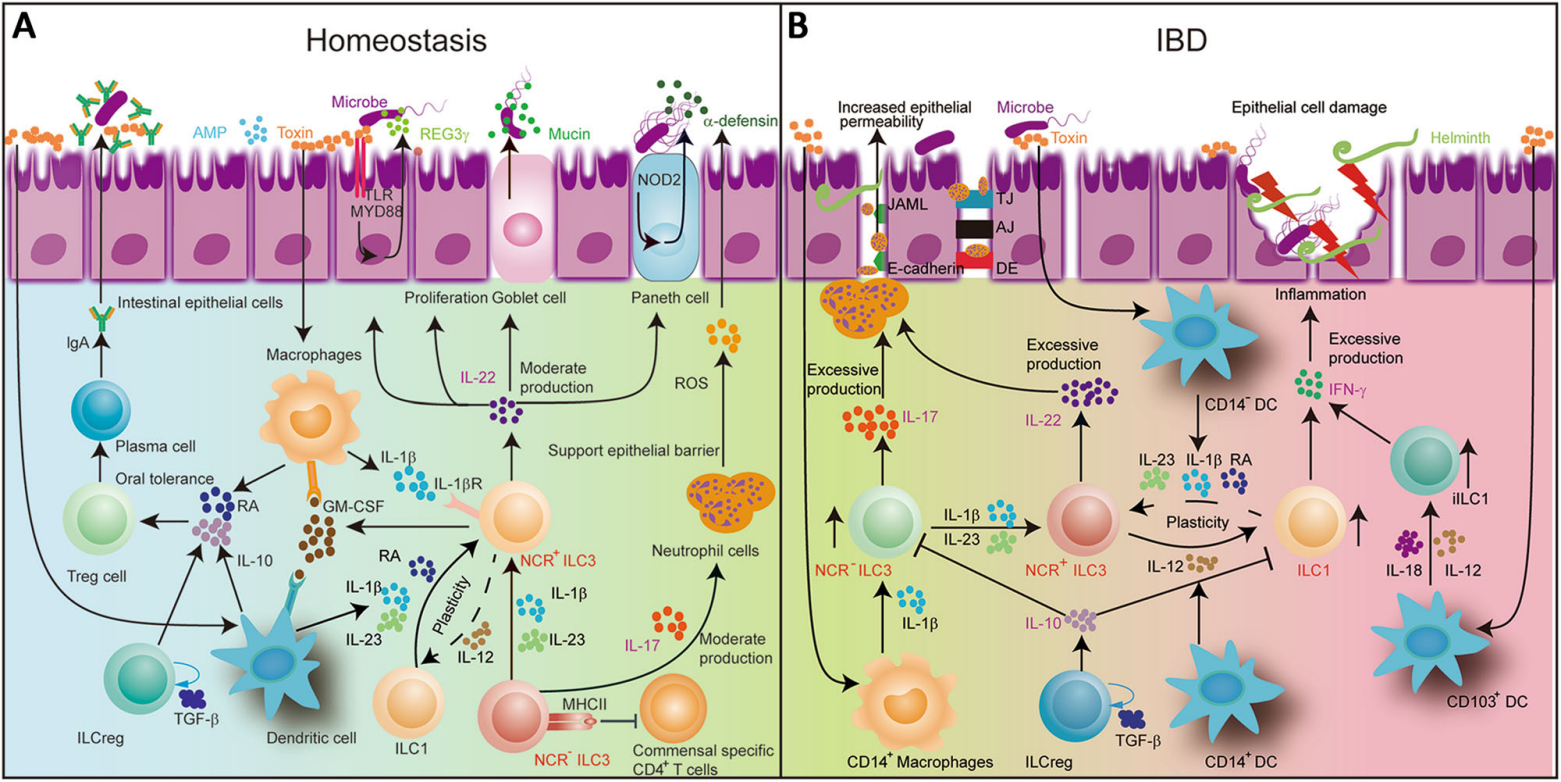
ILC3 in maintenance of gut homeostasis and occurrence of inflammatory bowel diseases. **a** Macrophages are stimulated by bacteria, releasing IL-1β. IL-1β engages an IL-1 receptor on ILC3, promoting IL-22, IL-17 and GM-CSF release. GM-CSF triggers DCs and Macrophages to generate retinoic acid and IL-10, which in turn promote the formation of Treg cells. IL-22 promotes epithelial barrier integrity and proliferation, inducing the production of AMPs, REG3γ and mucin. IL-17 can recruit neutrophils and also supports epithelial barrier protection. MHC-II-expressing ILC3 can inhibit commensal specific CD4^+^ T cells. NCR- ILC3 can switch to NCR + ILC3 with IL-1β plus IL-23 stimulation. **b** In IBD, the number of the IL-17-producing NCR^−^ ILC3 has been shown to be increased. IL-17 can recruit neutrophil cells. The neutrophil transmigration can disrupt junction proteins, such as E-cadherin and JAML, leading to the enhancement of epithelial permeability. The increase of the IFNγ-producing ILC1 cells of intraepithelial ILC1 and CD127^+^ ILC1 is accompanied by a large decrease in the number of NCR^+^ ILC3 cells. NCR^+^ ILC3 produces excessive IL-22 in IBD. ILC3 can differentiate into ILC1 under the stimulation of IL-12 produced by CD14^+^ DCs. This ILC3 to ILC1 plasticity is reversible in the presence of IL-23, IL-1β and retinoic acid produced by CD14^−^ DCs. The population of the IFNγ-producing ILC1 is increased at the cost of the decreased NCR^+^ ILC3 cells

IL-22, a member of the IL-10 family, displays a homologous secondary structure, binding to its heterodimeric receptors IL-22R1 and IL-10R2 on the surface of epithelial cells. IL-22 signaling induces the generation of mucin and pro-inflammation molecules. It also facilitates tissue repair through boosting epithelial cell proliferation and survival^49^. Besides, IL22 is able to promote the production of nucleotide oligomerization domain-containing protein2 (NOD2), which is related to the innate immune response. The activation of the NOD2 signaling can promote the secretion of mucin and AMPs protecting intestinal epithelial cells from invading bacteria^50^. Therefore, IL-22 contributes to preventing bacterial infections, relieving intestinal inflammation and restoring tissue injury during hepatitis or colitis (Fig. 2a)^51^. In a mouse model of graft-vs.-host disease, ILC3-derived IL-22 can activate intestinal stem cells to impede tissue damage^52^. In addition, ILC3 triggers intestinal epithelial fucosyltransferase 2 (Fut2) expression and fucosylation in mice through secreting IL-22 and lymphotoxin in a commensal bacteria-dependent or independent manner. Fut2 regulating H antigen expression in gastrointestinal mucosa has been reported to mediate the fucosylation of intestinal epithelial cells^53^.

Except for IL-22, the NCR^−^ ILC3 can also generate IL-17. Increasing evidence indicates that it can stimulate epithelial and endothelial cells to secrete chemokines and other chemoattractants, and also influence the inflammatory immune response by recruiting proinflammatory neutrophils^54^. The neutrophils play a protective role in supporting epithelial barriers and maintaining intestinal homeostasis through producing ROS and α-defensin (Fig. 2a). Recent researches demonstrate that γδ T cells are the source of early and protective IL-17 after an acute intestinal injury and IL-17 can maintain and protect epithelial barriers in the intestinal mucosa by regulating the tight junction protein in an independent-IL-23 manner^55^.

On the other hand, the interaction between macrophages and ILC3 can maintain gut homeostasis *via* GM-CSF. RORγt^+^ ILC3 are an important source of GM-CSF in the physiological state that relies on microbial signals and IL-1β production by macrophages^48^. IL-1β promotes ILC3 to produce GM-CSF, which initiates the release of retinoic acid (RA) and IL-10 by DC and macrophages in the mucosa to facilitate Treg proliferation^48^. GM-CSF mediates mononuclear phagocytes (MNP) that are composed of DC and macrophages to keep intestinal Treg balance (Fig. 2a). Deleting GM-CSF can alter the function of mononuclear phagocytes, leading to reduced Treg numbers and broken oral tolerance. CX3CR1^+^ MNP can produce IL-23 and IL-1β, dependent upon MyD88 signaling^56^. MNP detect microbial signals and present extracellular antigens to T lymphocytes.

In mice, ILC3 exert a crucial role in gastrointestinal mucosa immunity by directly promoting epithelial cell proliferation, inducing production of anti-inflammatory cytokines and antimicrobial peptides, preventing dissemination of intestinal bacteria, and suppressing CD4^+^ T-cell responses. Thus, we speculate that ILC3 might have similar functions in humans. ILC3 interact with CD4^+^ T cells in maintaining gut homeostasis and in response to stimuli from commensal bacteria. In fact, ILC3 can express MHC-II molecules in humans^47^. In mice, MHC-II^+^ ILC3 may present microbiota-derived antigens to commensal-specific CD4^+^ T cells (Fig. 2a). These commensal-specific CD4^+^ T cells are killed to induce tolerance to these commensals for lack of the co-stimulatory molecules CD40, CD80 and CD86^46,47^. However, IL-1β stimulates NCR^−^ ILC3 to produce CD80 and CD86 in mouse spleen^57^. This difference may be due to the different micro-environments in various tissues. MHC-II-producing ILC3 are found to regulate intestinal homeostasis by inducing apoptotic cell death-activated commensal bacteria-specific T cells^47^. It has been revealed that patients with pediatric Crohn’s disease have lower levels of MHC-II^+^ ILC3 than individuals without this condition^47^. Hence, ILC3 regulate intestinal homeostasis by a cytokine-dependent pathway and cell surface receptor regulatory mechanisms.

Strikingly, newly found ILCreg participate concomitantly in maintenance of gut homeostasis *via* secreting IL-10 and TGF-β. ILCreg noticeably inhibit the production of IFN-γ and IL-17A by ILC1 and NCR^−^ ILC3, respectively, to reduce their pro-inflammatory activity, while IL-22 production by NCR^+^ ILC3 is unaffected by ILCreg. In addition to IL-10, ILCreg can produce TGF-β1 in an autocrine manner, which supports the maintenance and proliferation of ILCreg. Therefore, if the inhibitory action of ILCreg is not enough to offset detrimental impacts produced by ILC1 and over-activated NCR^−^ ILC3 or NCR^+^ ILC3, inflamed intestinal lesions occur or are exacerbated.

## ILC3 in inflammatory bowel diseases

Using Rag2^−/−^Tbx21^−/−^ mice (TRUC mice), a mouse model of UC, Ermann et al. found that IL-23 triggered secretion of IL-17A by NCR^−^ ILC3 to play a significant role in the development of colitis^58^. Recent studies indicate that dysregulation of the IL-23/IL-17 axis is involved in various genetic susceptibilities in CD and UC patients due to dysfunction of the innate and adaptive immune responses^59^. IL-17 also acts on many types of stromal, epithelial and myeloid cells to generate a great number of pro-inflammatory cytokines, such as IL-1β, IL-6 and TNF, and chemokines enlisting neutrophils and macrophages. Emerging evidence shows that the increased level of migrating neutrophils can destroy junction proteins, such as E-cadherin and a junctional adhesion molecule-like factor, and produce microscopic gaps between epithelial cells, further impairing the epithelial barrier and exacerbating intestinal inflammation in IBD^60^. Moreover, massive neutrophil transmigration can alter the cellular expression levels of tight junction proteins, leading to injury of the epithelial barrier and enhancement of the epithelial permeability (Fig. 2b)^60^. In IBD, the number of CD68^+^ macrophages is abnormally increased. In the mucosa and submucosa, the infiltrating macrophages are found to express Toll-like receptors, such as TLR-2, TLR-4 and TLR-5^61^. However, secukinumab, a specific antibody against human IL-17A, fails to alleviate CD. On the contrary, it worsens this disease^62^ though IL-17A has a protective effect in the intestinal epithelial permeability^55^, suggesting that ILC3 do not impulse the pathological progress of the inflamed mucosa directly through IL-17A secretion.

The inappropriate activation of ILC3 has been proven to cause intestinal damage through excessive production of IL-22. This may induce epithelial cells to generate neutrophil chemoattractants, leading to accumulation of neutrophils and tissue destruction (Fig. 2b)^63^. Uhlig *et al* found that NKp46^+^ ILC3 could induce inflammation through the excessive production of IL-22 and GM-CSF in the anti-CD40 model of colitis^64^. Additionally, colonic ILC3 from UC and CD patients displayed a distinctly higher expression of IL-22 than in healthy individuals^56^. However, IL-22 was reported to have protective effects in some experimental models of colitis^65^ and reduced in CD patients^66^. In a Th2-mediated chronic colitis model (TCR□KO mice) representing UC, IL-22 could ameliorate intestinal inflammation by enhanced mucus production. IL-23-responsive IL-22 was also shown to relieve colon inflammation in murine colitis induced with dextran sodium sulfate or *Citrobacter Rodentium*^67^ and ILC3-deficient mice generated scant IL-22 to enhance sensitivity to *Citrobacter Rodentium*^68^. Niess et al. also found that the IL-22-deficient mice were highly susceptible to intestinal inflammation caused by *Candidiasis*^69^. Intestinal macrophages can accelerate the intestinal inflammation in CD patients through crosstalk with RORγt ILC3 to increase the production of IL-22. Another report demonstrates that CD14^+^ CX3CR1^+^ mononuclear phagocytes increase the expression of IL-22 in ILC3 through producing TL1A, IL-23 and IL-1β. DC are also able to mediate ILC3 to secrete IL-22^56^. Emphatically, although CD103^+^ DC and CD14^+^ macrophages may aggravate intestinal inflammation in CD patients, they are also able to facilitate a negative feedback pathway *via* the production of IL-22 by ILC3^70^. Collectively, IL-22 is a double-edged sword in intestinal inflammation and in intestinal tract protection.

The number of ILC1, especially in the lamina propria, is greatly increased to about 10–40% of the total ILC in the inflamed intestinal mucosa in CD patients^12,26,27^ with enhancing relative severity of the mucosa inflammation. The population of the IFNγ-producing ILC1 is increased at the cost of decreased NCR^+^ ILC3 in the inflamed intestine in IBD patients^12,26^. ILC3 can deviate towards ILC1 under the stimulation of IL-12 production by CD14^+^ DC^12^, suggesting the imbalance between ILC3 and ILC1 may result in CD. Bernink and his colleagues recently revealed that the differentiation of NKp44^+^ ILC3 to CD127^+^ ILC1 is reversible, relying on appropriate assembling of cytokines. For example, ILC1 may deviate into ILC3 in the presence of IL-23, IL-1β and retinoic acid produced by CD14^−^ DC^12^. The differentiation of IL-22-producing ILC3 into IFNγ-producing ILC1 has been proven to be highly associated with colitis development in mice (Fig. 2b). In some inflammatory situations, NKp46^+^ ILC3 down-regulate RORγt to promote T-bet expression, becoming a source of IFN-γ^71^. These cells are designated as ex-RORγt ILC3, participating in innate immune defense against infection by *Salmonella Thyphymurium*^30^. ILC2 generating IFN-γ and IL-13 have also been found in the intestinal tissues of CD patients^72^, suggesting a certain plasticity between ILC1 and ILC2 in response to IL-12. Recently, it has been indicated in Rorα-deficient and Rorα^sg/sg^/Rag1^−/−^ mouse models that the Rorα-dependent ILC3, rather than ILC2, function in the development of intestinal fibrosis, hinting at a potential therapeutic target for IBD^31^. Therefore, azathioprine and infliximab, an immunosuppressant and an antibody specific to TNF, respectively, are clinically harnessed to treat CD patients. A markedly decreased expression of IFN-γ was indeed observed in the inflamed gut mucosa, suggesting that IFN-γ might be a therapeutic option for CD. Of note, the CD patients treated with Fontolizumab, a humanized murine anti-IFN-γ antibody, did not show statistical efficacy in clinical trials although there was an improvement in clinical symptoms with a significant decrease in C-reactive protein levels^73^. These results indicate that IBD is a complicated pathological process mediated by multiple mechanisms. Single therapeutic strategy will difficultly operate upon this disease.

In the intestinal samples of patients with IBD, Geremia et al. found that Lin^−^ CD56^−^CD127^+^ ILC accumulated in the inflammatory ileum and colon of CD, but not UC patients. These cells could express IL17 and IFN-γ to respond to IL23 in vitro^74^. Increased ILC frequencies have recently been found in patients with primary sclerosing cholangitis-associated IBD, but not in those with UC^75^. Analysis of human tissue samples shows that plasticity between NCR^+^ ILC3 and ILC1 subsets is dependent on the local cytokine environment. The antimicrobial peptide-secreting-RORgt^+^NKp44^+^ ILC3 are trans-differentiated into ILC1 that produce IFNγ to induce chronic inflammation in the presence of IL-23 and IL-12^76^. This process was observed by the addition of IL-2 and IL-12 to culture fetal intestine NKp44^+^ ILC3. The combination of IL-2 with IL-12 rapidly caused the loss of NKp44 and c-kit expression in fetal intestine NKp44^+^ ILC3 with the acquirement of ILC1 phenotype^26^. Therefore, the population of IFNγ-producing ILC1 is increased at the cost of decreased NCR^+^ ILC3 cells in the inflamed intestine in IBD patients. Human gut ILC3 express not only a leukemia inhibitory factor (LIF) that stimulates proliferation of epithelial cells, but also IL-26 that negatively modulates proliferation of intestinal epithelial cells and facilitates ILC3 to produce proinflammatory TNF and IL-8^39^. CD14^+^ DC are source of IL-12 cytokines. They also secrete IL-22 binding protein that counteracts the role of IL-22^77^. These data may reflect ILC3 functional flexibility. In view of the above findings, Feagan et al. intravenously administered ustekinumab, a specific antibody to the p40 subunit shared by both interleukin-12 and interleukin-23, to treat patients with moderate to severe active CD. Consequently, the clinical remission in the patients was observed and if the ustekinumab was subcutaneously injected at a dose of 90 mg every 8 weeks or every 12 weeks, this relief could be maintained^78^, further supporting that the CD14^+^ DC-produced IL12 and the CD14^−^ DC-produced IL-23 indeed play a crucial role in the process of conversion of NCR^+^ ILC3 to ILC1.

Last but not least, a relationship of gut microbiota with IBD is concerned as well. Alteration of gut microbiota is closely linked to initiation or progression of IBD, but it is indistinct whether gut microbiota is a primary or secondary event. It is well known that a mutually beneficial symbiotic relationship between humans and gut microbiota is necessary for maintaining gastrointestinal homeostasis. However, intestinal flora is dynamically changing with age and environmental alteration^79^. Its composition and function is affected by various environmental factors, such as birth, diet, stress, antibiotic treatment and so on^80^. Among such environmental factors, diet appears to be an important modulator of intestinal immunity with direct or indirect effects on the structure and activity of the intestinal flora^81,82^. Short chain fatty acids (SCFA) are generated by the gut microbiota and regulated by patterns of food intake. Emerging evidence show that the effect of SCFA and their metabolite on IBD is mainly modulated by innate immunity responses and adaptive immune responses^83^. For example, Aryl hydrocarbon receptor (AHR) is present in intestinal epithelium, macrophages, B cells, T cells and dendritic cells. Kynurenine, an endogenous AHR ligand, is derivative of essential amino acid tryptophan, and many dietary ligands of AHR, including galangin, genystein, chrysin, apigenin and quercetin, belong to natural flavonoids residing in fruits and vegetables^84^. It has been proved that the diet-derived AHR ligands can mediate IL-22 expression to induce the generation of AMPs and mucin, thus protecting intestinal mucosa from pathogen invasion and maintaining barrier integrity^85^. On the contrary, the expression of innate-driven IL-22 was reduced for lack of AHR in ILC3, leading to the expansion of segmented filamentous bacteria with occurrence of colitis^86^. Collectively, these studies demonstrate that AHR acts as a necessary sensor for environmental factors and human lifestyle factors, such as diet, and is also essential for maintenance of NKp46^+^ ILC3 function.

Overall, ILC3 relies primarily on moderate production of IL-22, GM-CSF and IL-17 secreted by NCR^+^ ILC3 and NCR^−^ ILC3, respectively, to mediate the defense to pathogens. IL-1β and IL-23 with RA are released by macrophages and DCs under stimulation of microbe, which might keep the conversion of ILC1 to NCR^+^ ILC3 subset. The moderate production of IL-22 conduces to epithelial barrier integrity and proliferation, stimulating secretion of AMPs, REG3γ and mucin, and enhancing epithelial fucosylation. Alternatively, IL-17 can recruit neutrophil cells to support the protection of epithelial barrier by expression of ROS and α-defensin. GM-CSF triggers DCs and Macrophages to generate retinoic acid and IL-10, which facilitate the formation of oral tolerance. In addition, MHC-II-expressing ILC3 can inhibit commensal specific CD4^+^ T cells. If intestinal epithelia are continuously invaded by massive pathogens, IL-22 or IL-17 is overexpressed by NCR^+^ ILC3 or NCR^−^ ILC3. They recruit more neutrophil cells to excessively secrete pro-inflammatory factors, incurring the swift enhancement of epithelial cell permeability. Importantly, NCR^+^ ILC3 can deviate towards ILC1 under the stimulation of IL-12 generated by CD14^+^ or CD130^+^ DC. The IFNγ-producing intraepithelial ILC1 and CD127^+^ ILC1 are largely increased. The excessive production of IFNγ will result in epithelial cell damage, eventually exacerbating the inflammatory reaction.

## Therapeutic potential of ILC in inflammatory bowel diseases

As well known, IBD is a chronic non-specific inflammatory disease without effective drug treatment. At present, medical therapy focuses mainly on usage of anti-inflammatory drugs, such as thiopurines, mercaptopurine, 5-aminosalicylic acid and methotrexate (Table 2). In general, anti-inflammatory drugs are the first clinical practice in the process of IBD treatment to attenuate intestinal inflammation, but cause various adverse effects. Moreover, many patients with IBD do not procure clinical remission with the treatment of mesalazine, immunosuppressant and monoclonal antibodies against inflammatory cytokine TNF^87^. Thus, it is urgent to identify and develop novel drugs with high efficiency and low toxicity.

**Table 2:** Pharmacotherapy for IBD

Drug	Target	Mechanism	Reference
Aminosalicylates (5-aminosalicylates,Sulphasalazine, Olsalazine)	eIF4b, eIF4e	(a) Scavenging reactive oxygen species	^98, 99^
		(b) Upregulation of endogenous antioxidant systems	
		(c) Altering faecal bacteria profiles and exerting anti-inflammatory activities by inhibition of leukocyte motility	
		(d) Inhibiting tetrahydrobiopterin biosynthesis and NO formation	
		(e) Preventing mitochondrial damage by inhibition of phosphatidic acid formation and phosphatidylethanolamine degradation, and alteration of mitochondrial lipid composition	
		(f) Interfering with TNF-α, TGF-β, NF-κB and IL-1	
		(g) Suppressing the proliferation of human colon cancer cells and by inhibiting MMP-2 and MMP-9 expression *via* NF-κB-mediated cell signals and invasiveness	
		(h) Interacting with the Wnt/β-catenin pathway *via* inhibition of PP2A and with the active center of tumor suppressor PPAR-γ	
		(i) Arresting colon epithelial cells in S-phase by the activation of an ATR-dependent checkpoint and improving replication fidelity	
		(j) Down-regulation of expression of endostatin and angiostatin by modulation of MMP2 and MMP9 *via* inhibition of TNF-α	
Glucocorticoids (Budesonide, Hydrocortisone, Prednisolone)	undetermined	(a) Steroid-activated GR binds to glucocorticoid-responsive elements, resulting in modulation of antiinflammatory transcriptional pathways such as NF-κB, annexin1 and MAPK.	^100^
		(b) GR can decrease the expression of proinflammatory genes directly by protein–protein interactions.	
		(c) Glucocorticoids ameliorate ER stress in intestinal secretory cells by promoting correct protein folding and enhancing degradation of misfolded proteins.	
Immunomodulators (6-mercaptopurine, azathioprine, methotrexate)	undetermined	(a) Formation of thioguanine nucleotides leads to inhibition of DNA, RNA and protein synthesis, and induction of cytotoxicity and immunosuppression.	^101, 102^
		(b) Inducing T cell apoptosis by blockade of Rac1 activation upon CD28 co-stimulation and suppressing MEK, NF-κB, and bcl-xl	
		(c) Methotrexate competitively binds to folic acid in combination with dihydrofolate reductase, interfering with DNA synthesis and leading to cell death.	
		(d) Decreasing pro-inflammatory cytokine production and induction of lymphocyte apoptosis	
Antibiotics (Flagy, Ciprofloxacin, Cephalosporins)	undetermined	(a) Altering composition of intestinal bacteria, reducing harmful bacteria and promoting the growth of probiotics to reduce inflammation	^103, 104^
		(b) Reducing bacterial invasion of surrounding tissues in the intestinal lumen, and bacterial migration and systemic dissemination	
Biological agents (Infliximab, Adalimumab, Etanercept)	TNF-α	(a) Neutralizing the biological activity of TNFα by binding to the soluble and transmembrane forms of TNFα with high affinity, preventing it from binding to cellular receptors and inducing the lysis of cells	^105, 106^
		(b) Restoring the gut barrier, preventing leukocyte infiltration in intestinal mucosa and reducing the expression of β7 and CCR7 in leukocytes, thereby inhibiting inflammation	
		(c) Incurring apoptosis of T lymphocytes and mononuclear macrophage	

*eIF4b* eukaryotic translation initiation factor 4B, *eIF4e* eukaryotic translation initiation factor 4E, *NO* nitric oxide, *NF-κB* nuclear factor kappa-light-chain-enhancer of activated B cells, *PPAR-γ* peroxisome proliferator activated receptor-γ, *MMP-2* metalloproteinases 2, *PP2A* protein phosphatase 2A, *GR* glucocorticoid receptor, *MAPK* mitogen-activated protein kinase, *ER* endoplasmic reticulum, *Rac1* Ras-related C3 botulinum toxin substrate 1, *MEK* mitogen-activated protein kinase kinase, *bcl-xl* B-cell lymphoma-extra large, *CCR7* C-C chemokine receptor type 7

Over the past 10 years, ILC have gradually been recognized to be closely related to the pathogenesis of IBD and are promising to become an emerging therapeutic target. The transcriptional factor RORγt is indispensable for the development and differentiation of ILC3. Withers et al. used Rorc^flox^ and Id2^iΔROR-γt^ mice with ILC3 deficiency to establish an intestinal inflammation model through infection with *Citrobacter rodentium*. They found that the administered inhibitor of RORγt (GSK805) observably could relieve inflammation in mice by preserving ILC3 and limiting Th17 responses. The treatment of CD with GSK805 did not alter the proportion of ILC3, but reduced the percentage of pro-inflammatory Th17 and Th22 cells, suggesting that ROR-γt acts as a therapeutic target for IBD and other chronic inflammatory diseases^88^. In another aspect, IL-12 is implicated in playing a key role in ILC2 and ILC3 plasticity. It is a pro-inflammatory cytokine generated primarily by antigen presenting cells in answer to bacterial infection, and accumulated in the inflamed gut mucosa. Ustekinumab, a human specific antibody against p40, a subunit shared by both IL-12 and IL-23, can block IL-12 and IL-23 through binding an IL-12 Rβ1 receptor to relieve inflammation in patients with CD^89^. It can also induce and maintain the effects of anti-TNF therapy in moderate-to-severe CD patients^90^.

Many clinically used therapeutic agents may affect ILC differentiation, homeostasis or function. Target cytokine-cytokine receptors, such as IL-2-IL-2R, IL-12-IL-12R, IL-23-IL-23R, IL-1-IL-1R, TSLP-TSLPR and IL-6-IL-6R, play critical roles in the differentiation, function, and maintenance of ILCs. Targets 4β7 and MAdCAM-1 are responsible for the migration of ILCs, and targets TNF-TNFR and IL-17-IL-17R are implicated in the function of ILCs^91^. A developed strategy is to use the cytokines IL-33 or IL-25 to promote an anti-inflammatory ILC2 response, appearing effective in various chronic preclinical models of inflammation^92^. Another potential therapeutic approach is to facilitate the transition between the ILC1 and ILC3 subpopulations *via* the cytokines involved in ILC1/ILC3 plasticity^12^. In order to selectively regulate both protective and pathological ILC responses, some small molecule inhibitors of transcription factors, such as GSK805^88^, and other ILC modulators, including the vitamin A metabolite retinoic acid and Lipoxin A4^93^, are being developed. In addition, microbes and diet signals can greatly affect intestinal ILC^94^, which may also be an effective strategy to boost protective ILC responses while potentially limiting pathologic ILC responses.

## Conclusions

The detailed etiology and pathogenesis in IBD remain confused. However, the accumulating data indicate that the etiology of chronic intestinal inflammation is an inappropriate immune response to host microorganisms. Innate and adaptive immune responses may play a key role in the pathogenesis of IBD. Further evidence strongly supports that ILC3 maintain micro-environmental homeostasis of the gastrointestinal mucosa through moderate production of IL-22, IL-17 and GM-CSF to protect gut epithelia from microbe invasion in the physiologic state, but also contribute to the evolution and aggravation of IBD if IL-22 and IL-17 with IFN-γ become overexpressed due to dysregulation of ILC3 functions and with their transition towards ILC1 in the pathological state. Thus, ILC3 appear to be a double-edged sword in inflammatory bowel diseases. Even so, understanding of ILC3 is still in its infancy and many problems remain unclear. Thus, uncovering detailed roles of ILC3 in the various phases of the inflammatory immune responses is vital to elucidating the pathological mechanisms of IBD. The number of IL-17-producing NCR^−^ ILC3 is markedly increased in IBD. IL-17 can recruit neutrophil cells to disrupt E-cadherin and JAML, leading to the enhancement of epithelial permeability. IFNγ-producing ILC1 are enhanced at the cost of decreased NCR^+^ ILC3 levels. ILC3 can differentiate into ILC1 under the stimulation of IL-12 produced by CD14^+^ DC. This ILC3 to ILC1 plasticity is reversible in the presence of IL-23, IL-1β and retinoic acid produced by CD14^-^ DC. Hence, the antibody ustekinumab was conceived to block both IL-12 and IL-23 through binding to an IL-12Rβ1 receptor, ultimately relieving the clinical manifestation of IBD. However, other antibodies against human IL-17A or IFN-γ do not show significant efficacy in the treatment of CD patients, suggesting that the pathogenesis of IBD is highly complicated and a single therapeutic strategy will have difficulty operating upon this disease.
